# Morphology of the female reproductive system and spermatheca in *Notonecta maculata* Fabricius, 1794 (Heteroptera: Notonectidae)

**DOI:** 10.1515/biol-2025-1376

**Published:** 2026-10-01

**Authors:** Selami Candan, Nurcan Özyurt Koçakoğlu, Hakan Özdamar, Hicret Arslan

**Affiliations:** Department of Biology, Gazi University, Science Faculty, Ankara, Türkiye

**Keywords:** telotrophic meroistic type ovariole, vitellogenesis, chorion, spermatheca, light and electron microscopy

## Abstract

*Notonecta maculata* Fabricius, 1794 is an ecologically important freshwater predator of mosquito larvae and other aquatic invertebrates and has potential as a biological indicator of water quality. This study presents the first detailed microscopic anatomical and histological description of the female reproductive system of *N. maculata*, based on stereomicroscopy, light microscopy, and scanning electron microscopy. The female reproductive system consists of a pair of ovaries, each with seven telotrophic meroistic ovarioles, a spermatheca, and a genital chamber. Each ovariole is differentiated into a terminal filament, germarium, vitellarium, and pedicel. The germarium contains trophocytes, a trophic core, and young oocytes. The vitellarium includes approximately 14 oocytes at previtellogenic, vitellogenic, and choriogenic stages. During vitellogenesis, oocytes accumulate yolk granules, and the germinal vesicle shifts peripherally. In choriogenesis, the follicular epithelium flattens, and the chorion differentiates into endo-, exo-, and extrachorion layers. The pedicel is surrounded by a single-layered columnar epithelium and a thin muscle layer. The spermatheca comprises a reservoir and duct; the reservoir shows cuticular intima, glandular epithelium, and muscle layers, with mature sperm bundles in the lumen. These findings establish a baseline for future comparative, systematic, and reproductive biology studies.

## 1 Introduction

Insects, often the most abundant and diverse group of macroinvertebrates living in aquatic environments, have spread effectively throughout almost all aquatic environments [1]. Despite covering less than 1 % of Earth’s surface, inland waters are home to 10 % of all known animal species. Aquatic insects account for 60 % of these extant species, with approximately 100,000 currently described species [2].

Heteroptera Latreille, 1810 is an important part of the aquatic fauna that plays an essential role in the coastal food chain and is known to have indicator roles in their habitats [3], 4]. Aquatic and semi-aquatic Heteroptera species belonging to the infraorders Nepomorpha Popov, 1968, and Gerromorpha Popov, 1971 are distributed in almost every ecological niche with a variety of climatic conditions, including freshwater and marine habitats [5]. Back-swimming water bugs (Notonectidae Latreille, 1802: Nepomorpha) are invertebrate predators that may play a significant role in determining the abundance and structure of zooplankton populations in different freshwater habitats [6].

Heteroptera is a diverse group of insects with approximately 45,000 species worldwide, with up to 9,000 species distributed in the Palaearctic region [7], 8]. In Türkiye, there are 1,668 Heteroptera species. Forty-nine of these species belong to the Nepomorpha infraorder, while two genera and nine species belong to the Notonectidae (Nepomorpha) family [9], 10].

*Notonecta maculata* (Notonectidae) contributes to the maintenance of predator–prey balance by feeding on mosquito larvae and small aquatic organisms; this is particularly vital for the natural suppression of pest species such as mosquitoes [11], 12]. Additionally, members of the family Notonectidae are utilized as biological indicators due to their sensitivity to pollution and habitat degradation, thereby contributing to ecological assessments and water quality monitoring programs [13]. Due to these characteristics, *N. maculata* is considered a species of indirect economic importance, with potential for biological control, ecosystem services, and environmental monitoring. Therefore, the present study aims to describe for the first time the anatomical and histological structure of the female reproductive system of *N. maculata*, thereby contributing to a better understanding of its reproductive biology, physiological adaptations, and systematic and ecological significance.

## 2 Materials and methods

### 2.1 Obtaining insect samples

Aquatic insect specimens of the species *N. maculata* examined in this study were collected in February 2025 from the vegetation of a stagnant body of water located on the banks of the Kızılırmak River in Kırıkkale Province (Turkey), at coordinates 39°50′21.63″N, 33°28′25.40″E and an altitude of 683 m. Aquatic insect samples were collected in accordance with the TS EN ISO 10870 standard for the sampling of benthic macroinvertebrates in freshwater. Since the species is known to frequently inhabit areas with calmer water and abundant vegetation, sampling was carried out in this area using a scoop net (2 mm mesh size) [4], 14]. The collected specimens were brought to the zoology laboratory. In the initial stage, aquatic insect specimens that were believed to belong to *N. maculata* which is distinguished from other *Notonecta* species by its almost entirely red body with black spots were separated morphologically. However, to ensure accurate species identification, genital dissection was performed to confirm the correct taxonomic assignment aquatic insect specimens of the species *N. maculata* (n=10) [15], [16], [17], [18].

### 2.2 Stereo microscopy (SM) studies

All insect samples collected alive from the research area were brought to the laboratory in airtight, capped glass bottles on the same day and kept in aquariums in the Zoology Laboratory of the Biology Department of the Faculty of Science at Gazi University. The reproductive system of each specimen (*n* = 2) was completely dissected in buffered liquid (0.1 M sodium phosphate). Its morphology was described and photographed using a Stereo Microscope (SM) (Olympus SZX7).

### 2.3 Light microscopy (LM) studies

For histological examination of the structures of the female reproductive system of *N. maculata*, (*n* = 4) all structures were fixed with 10 % formaldehyde. All tissues were washed under running water and dehydrated with increasing levels of ethyl alcohol (from 70 to 99 %) before being embedded in paraffin blocks. Approximately 6 μm-thick tissue sections obtained from the tissue-embedded paraffin blocks were processed with Hematoxylin-Eosin (H&E) and Mallory’s Triple Stain to prepare permanent preparations. Histological processing followed standard paraffin histology procedures with minor modifications (19–21). Histological sections taken from various regions of the female reproductive system were examined with an Olympus BX51 Light Microscope (LM) and photographs of the necessary parts of the sections were taken.

### 2.4 Scanning electron microscopy (SEM) studies

Sample preparation for scanning electron microscopy followed conventional procedures for biological specimens [22], 23]. For scanning electron microscopy, cleaned samples (*n* = 4) were prepared in 0.1 M phosphate buffer (pH 7.2) and fixed with 2.5 % glutaraldehyde. After washing in the same buffer, the samples were dehydrated through a graded ethanol series (70–99 %). The specimens were then dried using Hexamethyldisilazane (HMDS) and mounted on stubs with double-sided tape. Subsequently, the organs were sputter-coated with a thin layer of gold using a Polaron SC 502 unit and examined with a Scanning Electron Microscope (JEOL JSM 6060 LV), at an accelerating voltage of 5 kV, and the images were documented [24].

## 3 Results

The female reproductive system of *N. maculata* is relatively long and linearly arranged, consisting of a pair of ovaries, a spermatheca, and a genital chamber (Figure 1a–c). Each ovary is composed of seven ovarioles attached apically to the abdominal wall by a terminal filament (Figure 1a and b). As oocyte development progresses, the oocytes become pale yellow in color, assume a cylindrical shape, and increase markedly in size compared with earlier stages (Figure 1a). Each ovariole can be clearly divided into four regions: the terminal filament, germarium, vitellarium, and pedicel. While the ovarioles are clustered together in the germarium region, they are freely arranged from the vitellarium to the pedicel, and a dense network of tracheae is evident between the ovarioles (Figure 1a).

**Figure 1:**
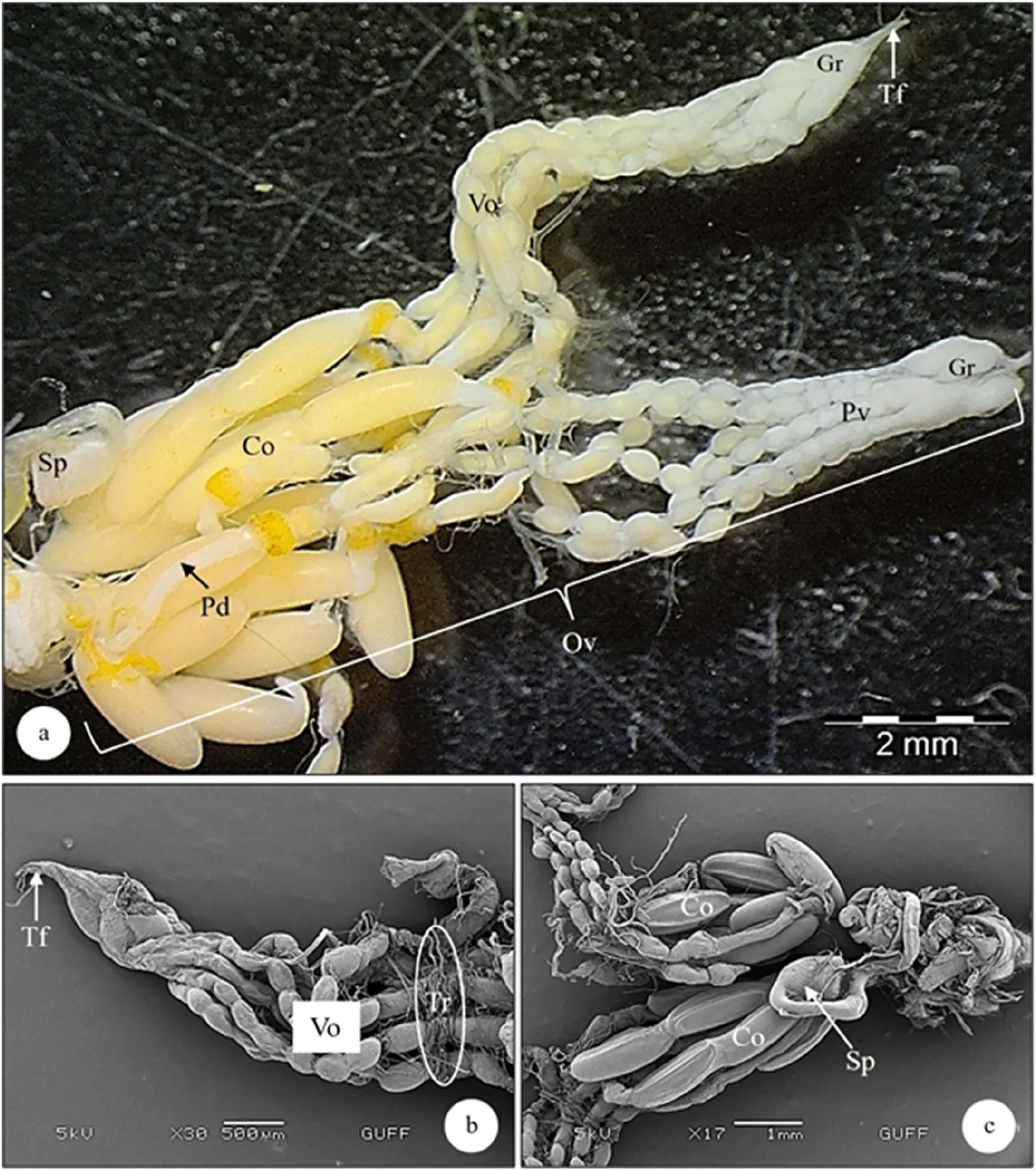
The female reproductive system. a. General view of the female reproductive system in *N. maculata* (SM). b. Ovarioles extending from an ovary (SEM). c. SEM micrograph of the ovary and spermatheca. Co, choriogenic oocyte; Gr, germarium; Ov, ovary; Pd, pedicel; Pv, previtellogenic oocyte; Sp, spermatheca; Tf, terminal filament; Tr, trachea; Vo, vitellogenic oocyte.

In histological sections and SEM micrographs of the germarium, which represents the second region of the ovariole following the terminal filament, trophocytes were observed, consistent with a telotrophic meroistic type of ovariole (Figure 2a and b). Moreover, histological sections of the germarium revealed a trophic core toward the central region, basophilic ooplasm toward the vitellarium, and young oocytes containing a centrally located germinal vesicle (Figure 2a, c, and d).

**Figure 2:**
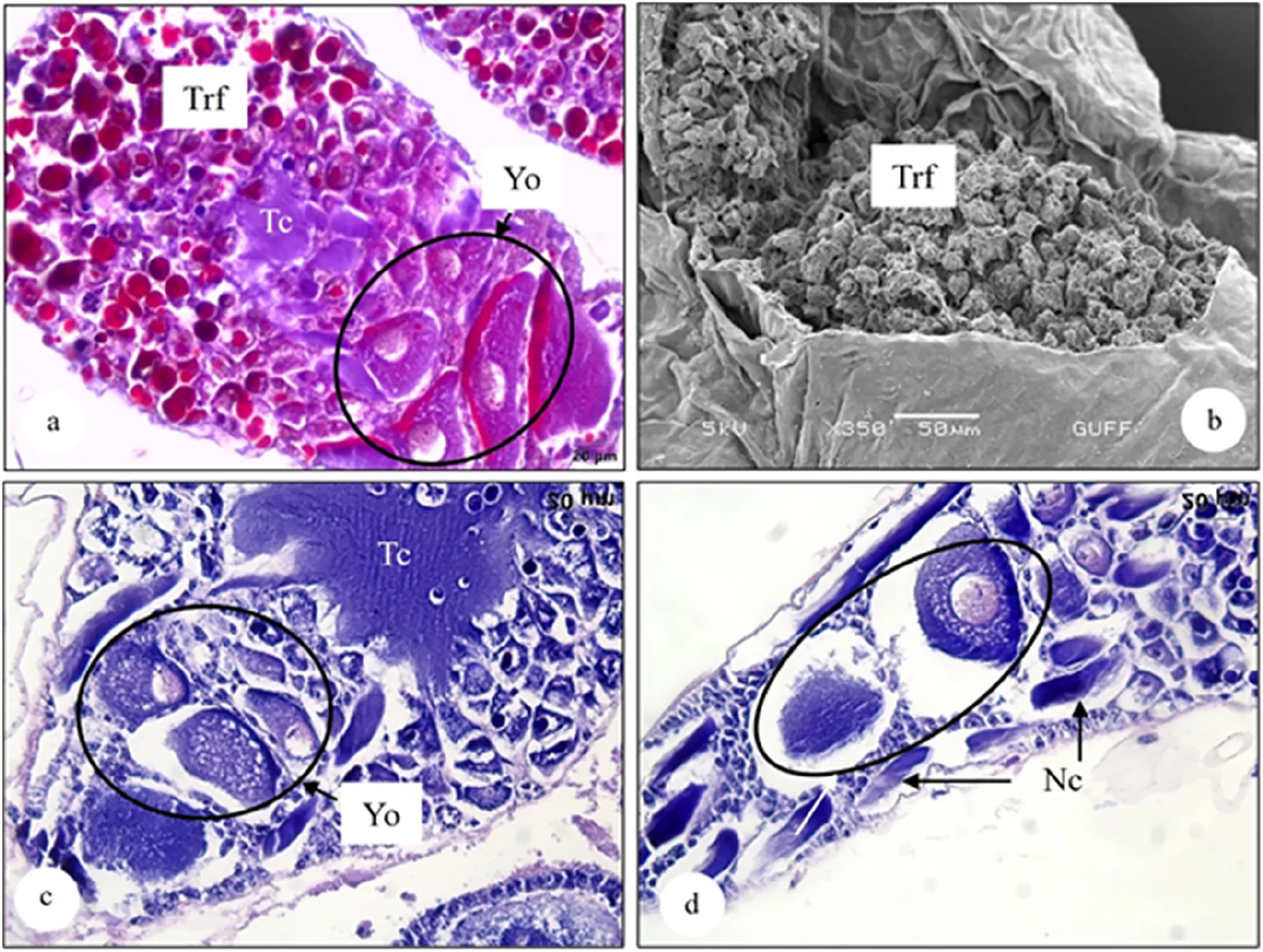
Germarium. a. Trophocytes, trophic core, and young oocytes in the germarium (Mallory stain) (LM). b. SEM micrograph of trophocytes. c. Histological section of the germarium showing the trophic core and young oocytes (H&E) (LM). d. Young oocytes and the nutritive cord in the germarium (H&E) (LM). Nc, nutritive cord; Tc, trophic core; Trf, trophocytes; Yo, young oocyte.

The vitellarium, which constitutes the third region of the ovariole, comprises approximately 14 oocytes undergoing three successive stages: previtellogenesis, vitellogenesis, and choriogenesis (Figures 1a, 3, 4). In the vitellarium, previtellogenic oocytes—the first stage of vitellogenesis—possess a single layer of columnar follicular epithelium surrounding the ooplasm, which lacks yolk granules (Figure 3a). At this stage, no distinct surface patterning is observed on the oocytes, whereas tracheal networks between adjacent oocytes are prominent (Figure 3b).

**Figure 3:**
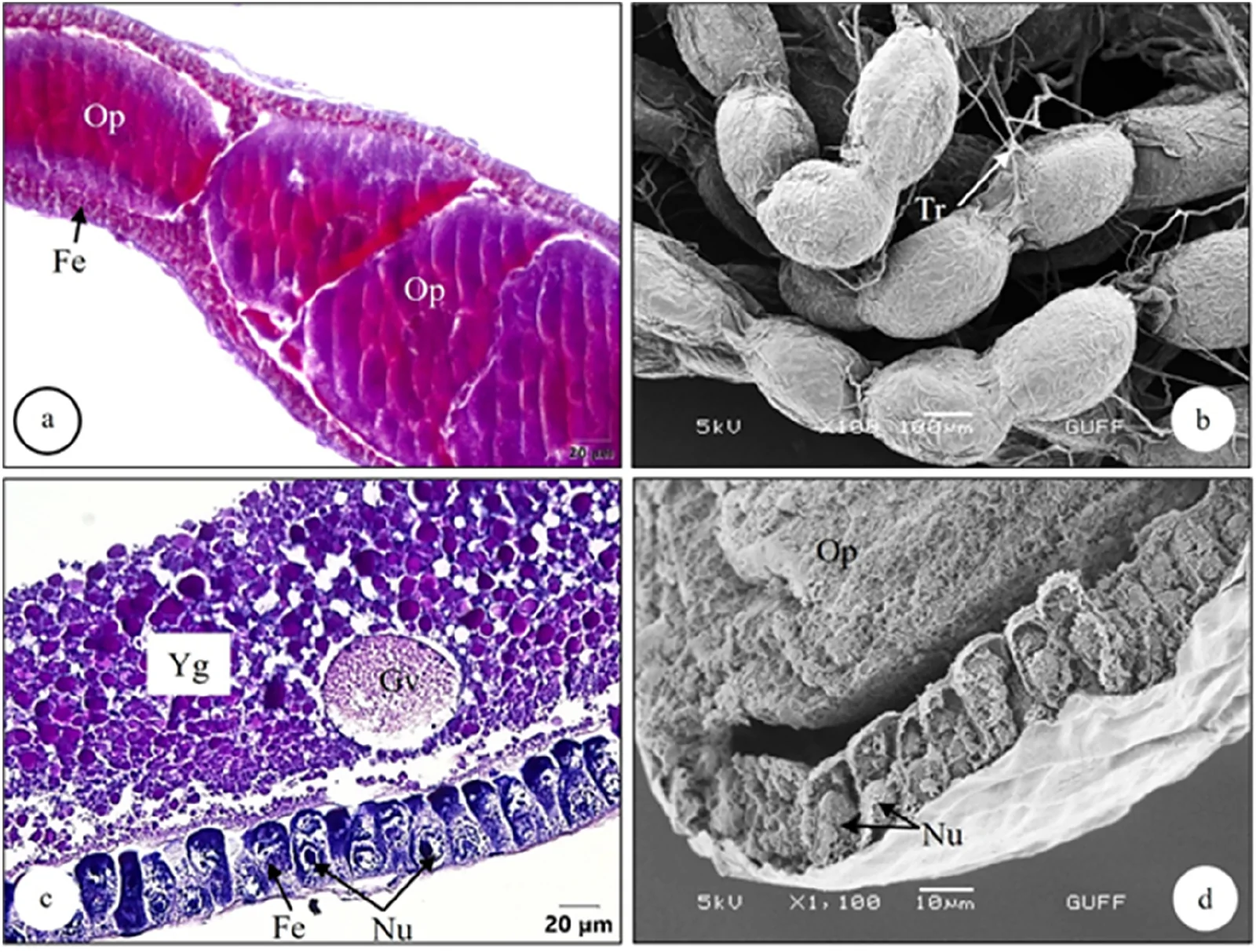
Previtellogenic and vitellogenic oocytes. a. Longitudinal section of previtellogenic oocytes (Mallory stain) (LM). b. SEM micrograph of previtellogenic oocytes. c. Histological section of a vitellogenic oocyte (H&E). d. SEM micrograph of a vitellogenic oocyte. Fe, follicular epithelium; Gv, germinal vesicle; Nu, nucleus; Op, ooplasma; Tr, trachea; Yg, yolk granules.

**Figure 4:**
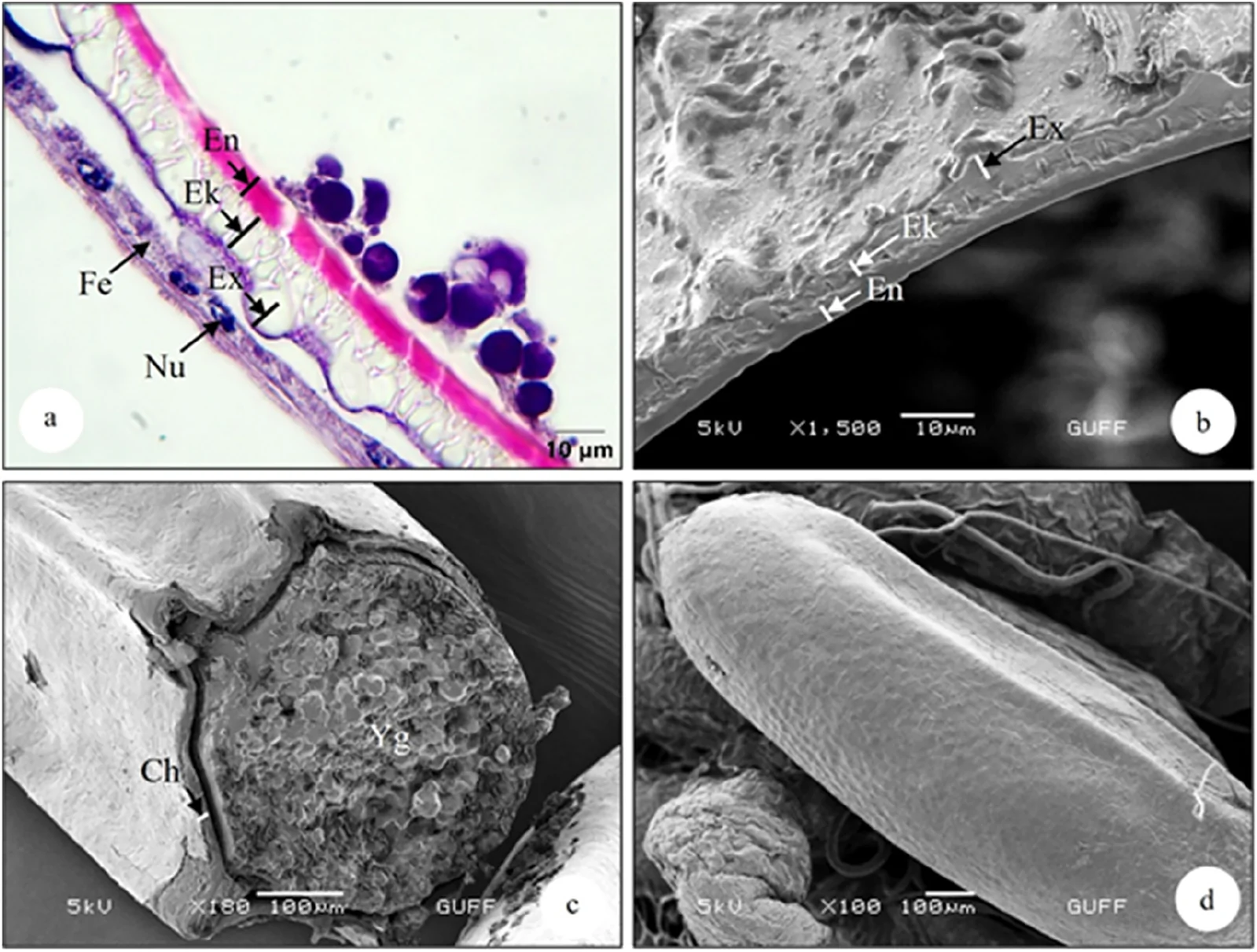
Choriogenic oocyte. a. Histological section of the chorion and follicular epithelium surrounding the choriogenic oocyte (H&E) (LM). b. SEM micrograph of the endochorion, exochorion, and extrachorion layers forming the chorion. c. Yolk granules in the ooplasm of the choriogenic oocyte (SEM). d. SEM micrograph of the surface of the choriogenic oocyte. Ch, chorion; Ek, exochorion; En, endochorion; Ex, extrachorion; Fe, follicular epithelium; Nu, nucleus; Yg, yolk granules.

During the second stage, vitellogenic oocytes exhibit ooplasm filled with yolk granules, displacement of the germinal vesicle toward the periphery, and a surrounding columnar follicular epithelium with nuclei located basally and characterized by a prominent nucleolus (Figure 3c and d). Near the transition from the vitellarium to the pedicel, two to three choriogenic oocytes are present; these oocytes are pale yellow, markedly larger than those at earlier stages, and cylindrical in shape (Figure 1a).

Histological sections of choriogenic oocytes show flattening of the follicular epithelium and its nuclei, accompanied by the formation of the chorion (Figure 4a). At this stage, the endochorion, exochorion, and extrachorion layers constituting the chorion can be clearly distinguished (Figure 4a and b). The ooplasm is densely filled with yolk granules (Figure 4c). SEM observations of choriogenic oocytes reveal a weakly defined surface pattern (Figure 4d).

The pedicel, which represents the distal region of the ovariole, is surrounded by a single layer of columnar epithelium with basophilic nuclei and a thin muscle layer, and exhibits luminal indentations (Figure 5a and b). The spermatheca, an organ of the female reproductive system, consists of a spermathecal reservoir and a duct. SEM observations of the reservoir reveal a morphology resembling the fruit of a eucalyptus tree (Figure 6a). In addition, a long, tubular duct with a wavy form connects the spermathecal reservoir to the common duct (Figure 6a). Longitudinal muscle fibers are evident on the surface of the spermathecal reservoir (Figure 6a). Histological sections and SEM micrographs of the spermathecal reservoir reveal a cuticular intima, a single layer of columnar glandular epithelium, and an associated muscle layer (Figure 6b and c). Aggregations of mature sperm bundles were observed within the lumen of the spermatheca (Figure 6d).

**Figure 5:**
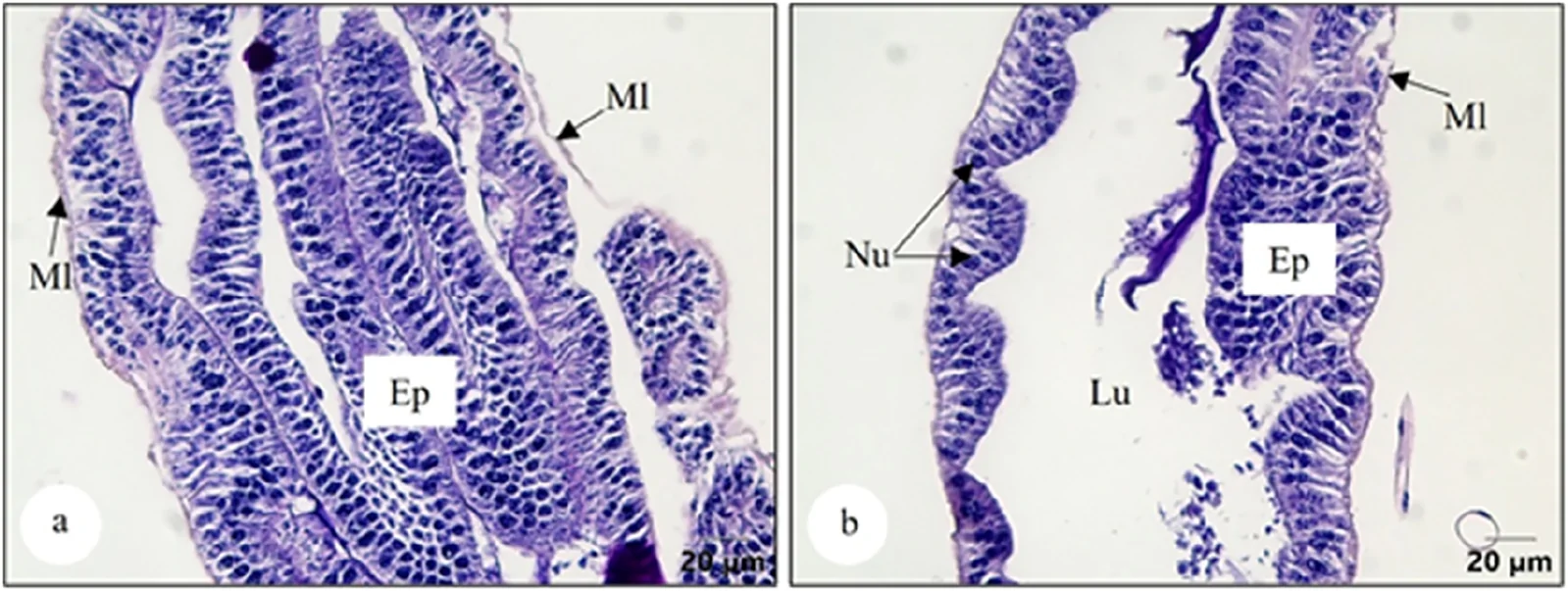
Pedicel. a, b. Longitudinal sections of the pedicel, the distal region of the ovariole (H&E) (LM). Ep, epithelium; Lu, lumen; Ml, muscle layer; Nu, nucleus.

**Figure 6:**
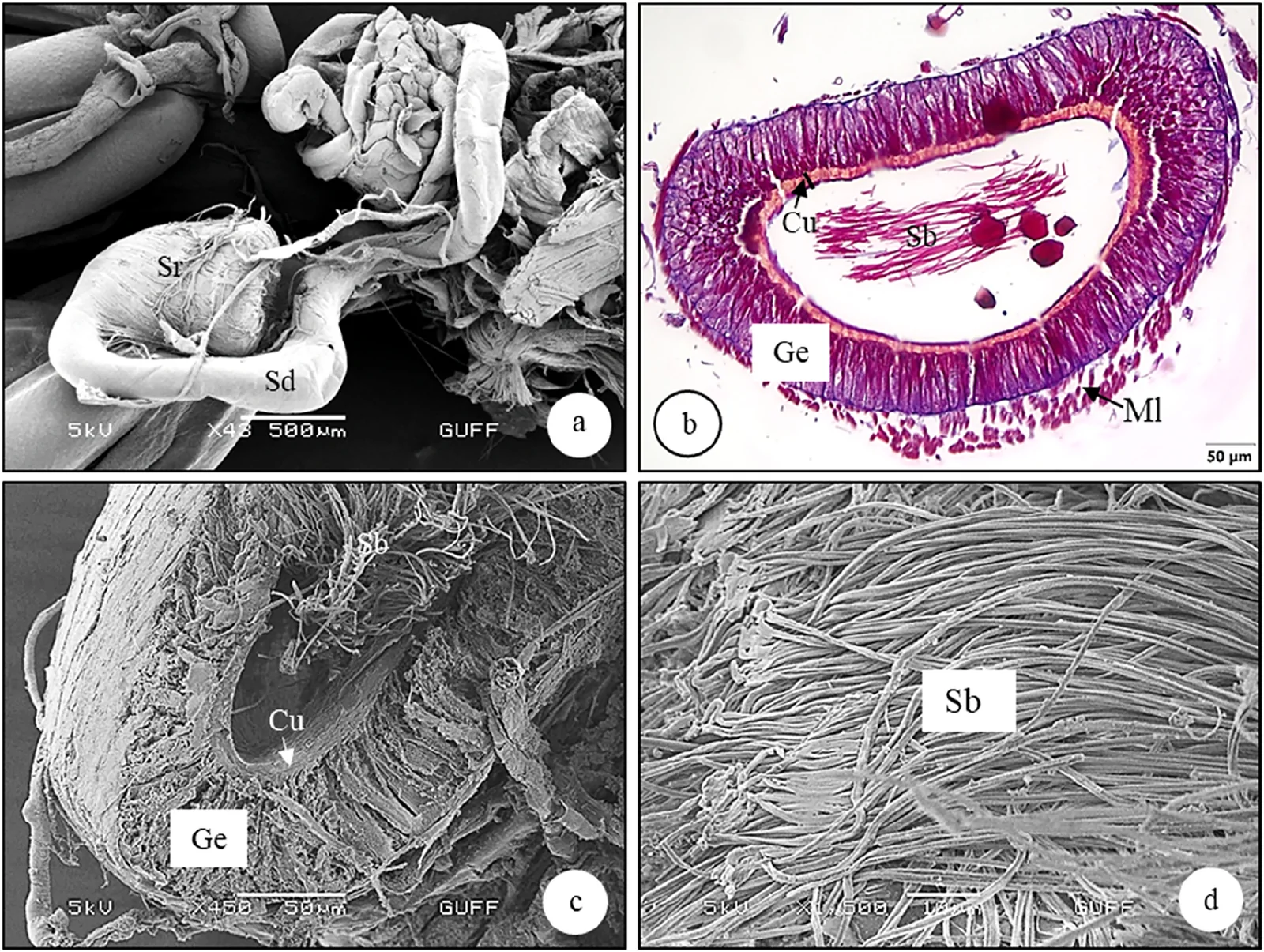
Spermatheca. a. SEM micrograph of the spermathecal reservoir and duct forming the spermatheca. b, c. LM (Mallory) and SEM micrographs of the cuticular intima and glandular epithelium surrounding the spermatheca. d. Aggregations of sperm bundles within the lumen of the spermatheca (SEM). Cu, cuticular intima; Ge, glandular epithelium; Ml, muscles layer; Sd, spermathecal duct; Sr, spermathecal reservoir; Sb, sperm bundles.

## 4 Discussion

The female reproductive system of *N. maculata* is consistent with the general structural organization reported for aquatic Heteroptera species, indicating that the reproductive system within this group has a conserved structure. Among Heteroptera families, the number of ovarioles in the ovary varies depending on the species. Four ovarioles have been reported in *Laccotrephes maculatus* (Nepidae) and *Plea minutissima* (Pleidae) [25]. *Belostoma*, *Diplonychus*, *Abedus*, and *Lethocerus* (Belostomatidae), *Aphelocheirus aestivalis* (Aphelocheiridae), *Nerthra macrothorax* (Gelastocoridae), *Laccotrephes japonensis*, *Nepa cinerea* (Nepidae) have five ovarioles. *Sigara*, *Cenocorixa*, *Corixa* (Corixidae), *Limnocoris*, *Pelocoris* and *Ilyocoris* (Naucoridae), *N. maculata* (Notonectidae), and *Ochterus marginatus marginatus* (Ochteridae) each consist of seven ovarioles [25]. On the other hand, six ovarioles are present in each ovary in the species *Hydrometra stagnorum* (Hydrometridae) [26]. The six to seven ovarioles found in species of *Notonecta glauca* (Notonectidae) are numerically consistent with our findings [27]. *Megamelus scutellaris* (Delphacidae) has been reported to have 11–13 tubular ovarioles in each ovary [28]. These differences among species indicate that the number of ovarioles is a variable reproductive trait associated with taxonomic diversity, reproductive capacity, and species-specific life strategies, rather than a fundamental difference in ovarian organization.

The telotrophic meroistic ovariol type identified in *N. maculata* shows similarities to the structures reported for *Abedus ovatus* (Belostomatidae) and *Gerris lacustris* (Gerridae) [29], 30]. Furthermore, the structural organization formed by the three-dimensional architecture of the ovariole and the tracheal network surrounding it is considered to support nutrient transport, oxygen delivery, and the orderly progression of oogenesis. Although the number of developing oocytes and the details of their developmental stages may vary among species, the developmental sequence observed in *N. maculata* is similar to the findings reported for *N. cinerea* (Nepidae) and *Halobates hayanus* (Gerridae) [24], 31]. In contrast, oocyte development in *A. ovatus* (Belostomatidae), is divided into seven stages [29]. Despite these differences, the fundamental developmental stages associated with oocyte maturation are considered to be conserved across Heteroptera species. The spermatheca is an ectoderm-derived organ connected to the anterior part of the common oviduct in most female insects. During mating, sperm are stored in this organ and can remain viable for extended periods until fertilization. The reproductive strategy is directly related to this function [32]. These findings indicate that females do not necessarily use sperm immediately after mating; instead, they can store it for extended periods and utilize it at an appropriate time. This behavior aligns closely with the functions of the spermatheca, including sperm storage, maintenance of sperm viability, and timed fertilization. The spermathecal structure in *N. maculata* is consistent with those described in *N. cinerea* (Nepidae) and *N. glauca* (Notonectidae) [24], 27].

## 5 Conclusions

In conclusion, this comprehensive characterization of the female reproductive system of *N. maculata* provides valuable insights into the reproductive biology of the Notonectidae family. The stability in the number of ovarioles, the stages of oocyte development, and the morphology of the spermatheca are findings that reinforce the species’ adaptive success in aquatic ecosystems and its taxonomic position. It is anticipated that the findings obtained from this study, when evaluated from a new and comprehensive perspective, will form a fundamental theoretical and methodological foundation for illuminating the reproductive biology of aquatic species in the future.

## 6 Study limitations and future perspectives

This study provides a descriptive histomorphological characterization of the female reproductive system and spermatheca of *N. maculata.* Accordingly, quantitative image analysis, functional histochemical investigations, and the evaluation of paracrine distribution patterns were beyond the scope of the present study. The application of these approaches will contribute to a more comprehensive understanding of the functional organization of the reproductive system and should be addressed in future studies using histochemical, complementary, and quantitative image analysis techniques.
